# Detection of circulating antigens for *Taenia* spp. in pigs slaughtered for consumption in Nairobi and surroundings, Kenya

**DOI:** 10.1016/j.parepi.2019.e00093

**Published:** 2019-02-07

**Authors:** James M. Akoko, Ewan MacLeod, Lian F. Thomas, Pablo Alarcon, Erastus Kang'ethe, Velma Kivali, Dishon Muloi, Patrick Muinde, Maurice K. Murungi, Julius M. Gachoya, Eric M. Fèvre

**Affiliations:** a International Livestock Research Institute, Nairobi, Kenya; b Division of Infection and Pathway Medicine, Deanery of Biomedical Sciences, College of Medicine and Veterinary Medicine, University of Edinburgh, Edinburgh, UK; c Royal Veterinary College, University of London, London, UK; d University of Nairobi, Nairobi, Kenya; e Institute of Infection and Global Health, University of Liverpool, Liverpool, UK; f Centre for Immunity, Infection and Evolution, University of Edinburgh, Edinburgh, UK; g Usher Institute of Population Health Sciences & Informatics, University of Edinburgh, Edinburgh, UK; h Directorate of Veterinary Services, State Department of Livestock, Ministry of Agriculture, Livestock and Fisheries, P.O Private Bag, Kangemi 00625, Nairobi, Kenya; i Maseno Univerisy, Maseno, Kenya

**Keywords:** *Taenia* spp., Porcine cysticercosis, Epidemiology, Food safety, Value chain, Kenya

## Abstract

**Background & methods:**

*Taenia solium* a zoonotic tapeworm, responsible for neurocysticercosis in humans is a major public health threat, being a leading cause of acquired epilepsy in endemic regions. Eastern and southern African nations have experienced a recent rapid growth in pig production, including small-scale, free-range systems, with an accompanying increased risk of *T. solium* transmission. Seven hundred blood samples were collected from randomly selected pigs presented for slaughter at one of the largest porcine abattoir supplying unprocessed pork to Nairobi city and its surroundings. The samples were tested using an antigen ELISA to determine the prevalence of infection with *Taenia* spp.

**Results:**

The prevalence, adjusted for diagnostic test characteristics, was estimated to be 4.4% (95% CI: 1.9–7.1) with no significant statistical difference by pig sex or age. Infection with *Taenia* spp. was detected in pigs from all regions of the country supplying pigs to this slaughterhouse. Official post-mortem inspection did not detect cysticercosis in the duration of the study. Therefore, all the carcasses entered the food chains of Nairobi (70%), or neighboring counties (30%).

**Conclusions:**

Circulating antigens of *Taenia* spp. were detected in pigs slaughtered in one of the largest porcine slaughterhouses in Kenya, which receives pigs from several regions in the country. This is an indication that pigs entering the value chain are raised under poor husbandry conditions and that pork consumers in Nairobi and its surroundings may be exposed to the important zoonotic parasite. Whilst further research utilizing full carcass dissection is required to confirm *T. solium* positive cases, interventions to improve food-safety throughout the pork value chains in Kenya should be seriously considered.

## Highlights

• *Taenia* spp. were detected in pigs (4.4%) slaughtered for Nairobi’s pork market.
• *Taenia* spp. infections were present in pigs from each region in Kenya supplying pork to Nairobi during this study
• A comprehensive risk assessment is necessary to identify exposure to food-safety hazards in pork value chain
• Proper cooking of pork is recommended, as a way of reducing the risk of transmission of *Taenia* spp. to consumers
• Interventions to improve food-safety should consider the full pork value-chain

## 1 Introduction

*Taenia solium*, a zoonotic tapeworm, the etiological agent of neurocysticercosis, has the greatest burden of any foodborne parasite and is considered one of the neglected tropical diseases (Torgerson and Macpherson, 2011; Diseases, U.t.C.N.T., 2012). It is endemic in Africa, Asia and Latin America, where poor sanitation and close human and pig interaction is common (Carabin et al., 2017). Porcine cysticercosis, human cysticercosis and taeniosis have all been previously reported in Kenya (Eshitera et al., 2012; Fèvre et al., 2017; Thomas et al., 2015; Wardrop et al., 2015) and cysticercosis has been identified as a priority zoonoses in this country (Munyua et al., 2016). A national scale assessment of prevalence and an understanding of the transmission dynamics, has however, yet to be carried out. Nairobi city has two principal sources of pork supply. First, a large integrated company, which controls the full chain from breeding units, fattening units, slaughtering, processing and supply, supplies pork products to the higher end domestic market and for export. The second major source of pork products is through two large independent pig abattoirs. These are abattoirs which serve as a congregation of multiple pig and pork sellers, buyers and privately employed butcher representatives. People working in the abattoir largely operate independently of each other, and the meat is largely sold in the small retailers or directly to consumers.

This study aimed to estimate the prevalence of *Taenia* spp. infection in pigs entering the food chain of Kenya's capital city through one large independent abattoir.

## 2 Materials and methods

The study was conducted in one of the largest independent pork abattoirs in Kenya (hereafter referred to as the study facility), which slaughterers approximately 60–90 pigs a day. This facility obtains pigs predominately from small scale farmers across several regions of Kenya and then supplies unprocessed pork to butcheries in Nairobi and its surrounding areas (Murungi et al., 2015).

Individual interviews were carried out with pig traders and farmers present at the abattoir using a structured questionnaire, to obtain information regarding the source (including county of origin) of the sampled pigs. Observations were made by the sampling team as to whether carcasses or organs were condemned due to the presence of cysticercosis or other causes. In addition, the government meat inspector positioned at the facility provided a weekly update on the detection of cysticercosis amongst slaughtered pigs during routine meat inspection. Copies of the movement permits and certificates of transport were obtained from the meat inspector at the abattoir to determine the sources of pigs and destinations of pork respectively.

All pigs presented for slaughter at the study facility between the months of October and December 2014 were considered eligible for sampling. To obtain an adequate sample size for the estimation of population prevalence with a 95% confidence level and a precision of 5%, a 32.8% expected prevalence rate (Eshitera et al., 2012) was used, based upon the most recent data available from Kenya at the time of study design. A design effect of 2 was assumed to cater for the potential clustering of pigs based on the different sources of origin. The minimum sample size required was calculated as 678 (Cochran, 1977), which was rounded up to 700 pigs. A systematic random sampling method was used to select pigs for sampling. The first pig presented for slaughter was sampled, followed by every fifth. An average of 15 pigs were sampled daily, over a period of 47 days. Pig snares were used for restraint and blood collected from the cranial vena cava into a labeled 10 ml serum tube using a BD Vacutainer® needle. The samples were transported in a cool box with ice packs to the International Livestock Research Institute (ILRI), Nairobi, within 5 h of collection.

Samples were centrifuged at 2500 rpm for 20 min. Serum was aspirated using a sterile disposable pipette and transferred into cryo-tubes, labeled with the individual pig barcodes, then stored at −80 °C for testing at a later date.

A commercial enzyme-linked immunosorbent assay kit, the apDia cysticercosis Antigen (Ag) ELISA (*apDia Turnhout*, *Belgium*), was used to detect circulating antigens for *Taenia* spp. in porcine sera samples that were tested in duplicate according to the manufacturer's guidelines, with the cut-off value calculated per plate as twice the mean OD of the negative control.

The R statistical software version 3.0.2 (R Core Development Team, 2008) was used to estimate the true prevalence, estimated using the package ‘prevalence’ (Devleesschauwer et al., 2013), which accounted for test sensitivity (86.7%) and specificity (94.7%) (Dorny et al., 2004),

## 3 Results

Seven hundred pigs were sampled in this study: pigs were sourced from eleven different counties, with the majority being sourced locally, coming from Kiambu (59.7%) and Nairobi (22.7%) counties respectively. During the study period, the majority of pork was distributed within Nairobi county (70%), with the remaining proportion distributed to Nakuru, Kajiado, Narok, Lakipia and Kwale counties. A large proportion (54%) of the pork destined for Nairobi was sold to a wholesale meat market located in the central business district, from where it was extensively distributed. The rest was distributed directly to several small-scale butcheries and restaurants that are predominantly located in low and middle income areas across the city.

Sixty-one pig samples tested positive for *Taenia* spp. by Ag-ELISA with an estimated true prevalence of 4.4% (95% CI: 1.9–7.1). *Taenia* spp. was detected from pigs sourced from all regions supplying pigs to the study facility during the sampling period, with the highest prevalence being found in pigs coming from Western and the lowest prevalence from Nairobi county (Table 1).

**Table 1:** Prevalence of *Taenia* spp. as detected by Ag-ELISA in pigs slaughtered at one of the largest porcine slaughter houses in Kenya.

Region	County	No of positives	No of samples	Apparent prevalence	True prevalence
Western	Bungoma	0	9	0	12.8 (4.5–22.6)
	Kakamega	0	18	0	
	Kisumu	0	1	0	
	Homa Bay	9	48	18.8	
	Migori	4	11	36.4	
	Total	13	87	11	
Central	Kiambu	37	418	8.9	4.6 (1.5–8.2)
	Embu	0	3	0	
	Nyeri	1	6	16.7	
	Total	38	427	8.5	
Rift Valley	Nakuru	1	6	16.7	16.7 (4.7–44.8)
	Kajiado	1	6	16.7	
	Total	2	12	16.7	
Nairobi	Nairobi	8	159	5	2.0 (0.1–6.0)
Total		61	700		4.4 (1.9–7.1)

Official post mortem meat inspection by palpation and incision, did not detect cysts in any of the pigs slaughtered during the entire project period. No carcass or organ was condemned for any reason (*Taenia* spp. infection or otherwise) during the course of 47 days of data collection.

## 4 Discussion

This study estimates the prevalence of *Taenia* spp. in pigs that are serving the growing demand for pork in the city of Nairobi. The estimated true prevalence of 4.4% (95% CI: 1.9–7.1) is similar to that more recently estimated within two smaller pig slaughter facilities within the same region of 4.35% (95% C.I. 2.5–7.4) (Nguhiu et al., 2017).

Meat inspection, carried out by the one government meat inspector stationed at the facility, did not find *T. solium* or *T. hydatigena* infections in any of the pigs slaughtered during the sampling period. Meat inspection is renowned for having low sensitivity for the detection of *T. solium* especially in light and moderate infections (Dorny et al., 2004). Examination of meat inspection records alone would therefore seriously underestimate the prevalence of porcine cysticercosis. Traditional meat inspection also involves making several incisions in meat that may affect the quality and safety through incision – mediated cross contamination of meat, visual inspection is therefore being considered in some countries where sufficient food-chain-information is available (Fredriksson-Ahomaa, 2014).

Although serological tests have been reported to provide better sensitivity than meat inspection techniques, they are not capable of differentiating *Taenia* spp. (Dermauw et al., 2016). Serological results, therefore require caution in their interpretation, where *T. hydatigena* is also present being that only *T. solium* is of public health concern (Lightowlers et al., 2016). Previous studies in Ghana and Tanzania suggest that *T. hydatigena* prevalence ranges between 1.4% and 6.7% (Braae et al., 2015a; Permin et al., 1999; Ngowi et al., 2004). Despite these diagnostic uncertainties in epidemiological studies in the field, given the burden of cysticercosis in humans and the lack of knowledge of its transmission in potentially endemic areas, it remains of value to apply serological tests to describe, with the inherent uncertainties, the extent of infection in the community and in the food chain.

Regardless of the species of *Taenia* detected within this population of slaughter pigs, one message that can be learnt from these results is that pigs entering the food chain are likely being raised under inadequate husbandry conditions. *T. hydatigena* requires a canid definitive host, with infective eggs shed in faecal material and being ingested by the intermediate host whilst grazing (Scala et al., 2015). The highest prevalence was identified in pigs coming from the western region (The number of pigs from the Rift Valley were too small to make an accurate assessment). This is unsurprising given the types of pig production systems in western Kenya where many pigs are kept under free-range, scavenging, conditions (Kagira et al., 2010; Wabacha et al., 2004; Mbuthia et al., 2015; Thomas et al., 2013). These systems increase the risk of exposure to *Taenia* spp., through the consumption of infective eggs in human or canid faecal material. *Taenia* spp. eggs may also be consumed by pigs from contaminated, feed sources, as suggested by Braae et al. who identified feeding potato waste to being as a risk factor for *Taenia* spp. infection (Braae et al., 2015b). The lowest prevalence of *Taenia* spp. detected was in pigs from Nairobi county, where we know that the majority of pigs are confined, therefore with low opportunity to consume human or canid faecal material (Wabacha et al., 2004). It is worth noting that the prevalence of *Taenia* spp. infections in pigs from Kiambu county was also concerning high, considering the large number of pigs sourced from this county, suggests that pig-production practices in this county require investigation.

Free-ranging pigs, or those raised under semi-intensive conditions receiving household or commercial waste as feed, are also at increased risk of being exposed to toxoplasmosis, trichonellosis, gastrointestinal nematode infections and African swine fever (ASF). These systems are also characterised by poor production performance and low profitability, as well as the potential risk for theft and injury of pigs (Carter et al., 2013; Levy et al., 2014; Nantima et al., 2015; Lekule and Kyvsgaard, 2003).

Interventions to improve pig production systems and associated value chains in Kenya are needed to mitigate public health risks as well as to increase the economic benefits to the value chain actors (Lekule and Kyvsgaard, 2003; Jaffee et al., 2019).

A recent study, describes a cross-sectional study within a county neighboring Nairobi and reports a prevalence of *T. solium* taeniosis in people of 6.8% as detected by microscopy with the staining and counting of uterine branches in identified proglottids (Mbuvi, 2018). Such information together with the findings of this study indicates that there is a very real likelihood that *T. solium* is entering the pork value chain through the study facility.

In order to better assess the food-safety hazards arising from pork consumed in Kenya, we recommend that a quantitative risk assessment is conducted, considering a multitude of foodborne pathogens. This will incorporate the identification of high-risk counties, and could potentially inform a risk-based meat inspection, whereby pigs from these areas may be subjected to periodic partial carcass dissection. Partial carcass dissection, involving dissecting the tongue, heart and masticatory muscles, has been found to be effective in detecting about 80% of both light and heavily infected pigs (Lightowlers et al., 2015).

At present there are no routine post-harvest interventions to prevent *T. solium* entering the food chain such as the freezing, salting or pickling of pork (Sotelo et al., 1986; Rodríguez-Canul et al., 2002), leaving proper cooking by consumers as the key strategy by which cysticerci may be inactivated. Unfortunately, the situations in which pork is consumed may be contributing to the risk of consuming poorly cooked meat.

As has been seen in neighboring Uganda, urban areas in Kenya have seen a rapid increase in pork being consumed at so-called ‘pork joints’ where pork is often consumed alongside alcohol. (Tatwangire, 2013; Kungu et al., 2017). Beliefs that alcohol in the stomach can kill any worms consumed in pork through ‘making the worms drunk’, as well as a perception that the fat in undercooked pork absorbs alcohol and thereby allow one to drink more, exposes customers of such establishments to the risk of consuming poorly cooked, and therefore potentially infective, pork products (Thompson, 2017). Public health educational programmes to ensure proper cooking of pork in households and restaurants are recommended to mitigate against the potential risk of exposure to both *T. solium* and other foodborne pathogens.

## 5 Conclusion and recommendations

This study found evidence of *Taenia* spp. infection in pigs slaughtered at one of the largest independent abattoirs in Nairobi. Although we are limited by the lack of species specific serological tests, it is possible that at least some, if not all, of these seropositive pigs are infected with *T. solium,* representing a public health risk to pork consumer in Nairobi and environs. Currently, no pre or post-harvest control measures for this parasite are in place and prevention of infection depends on proper cooking of pork meat at the consumer level.

The results presented here indicate that pigs entering the food chain in Nairobi from several regions of the country are being raised under unsanitary conditions, exposing them to infection with *Taenia* spp. Poor pig husbandry represents a public health, economic and animal welfare problem and a value chain approach to mitigate foodborne risks, whilst improving the profitability of the pork produced in the region, should be considered.

## Ethical considerations

The project was reviewed and approved by the ILRI Institutional Animal Care and Use Committee (IACUC Ref No. 2014.34). The Director of Veterinary Services and the County Director of Veterinary Services also approved and supported the study. At the abattoir, written informed consent was sought before conducting interviews and sample collection.

## Availability of data and materials

The datasets used in this study are available on the University of Liverpool Data Repository http://dx.doi.org/10.17638/datacat.liverpool.ac.uk/669.

## Competing interest

The authors have declared that no competing interest exist.

## Funding

This study was supported by the Medical Research Council (UK), Biotechnology and Biological Science Research Council (UK), the Economic and Social Research Council (UK), the Natural Environment Research Council (UK), through the Environmental & Social Ecology of Human Infectious Diseases Initiative (ESEI), Grant Reference: G1100783/1. This work also received support from the CGIAR Research Program on Agriculture for Nutrition and Health (A4NH), led by the International Food Policy Research Institute (IFPRI). We also acknowledge the CGIAR Fund Donors (https://www.cgiar.org/funders). James M. Akoko is currently supported by the Afrique One - African Science Partnership for Intervention Research Excellence (ASPIRE), under the Africa Institutions Initiative funded fully by the Wellcome Trust and the DELTAS Africa Initiative [Afrique One-ASPIRE/DEL-15-008]. Afrique One-ASPIRE is funded by a consortium of donor including the African Academy of Sciences (AAS), Alliance for Accelerating Excellence in Science in Africa (AESA), the New Partnership for Africa's Development Planning and Coordinating Agency (NEPAD), the Wellcome Trust [107753/A/15/Z] and the UK government. The funders had no role in the study design, data collection, analysis or interpretation, the writing of the manuscript or the decision to submit the manuscript for publication.

## Authors' contribution

1. Conceptualization: AJM EMF.
2. Formal analysis: AJM EMF LTF.
3. Funding acquisition: EMF KEK.
4. Investigation: AJM MKM MP KV MD.
5. Methodology: AJM EMF EM PA.
6. Project administration: AJM EMF.
7. Supervision: EMF EM KEK.
8. Writing – original draft: AJM & LFT.
9. Writing – review & editing: all authors.
